# Urban visual pollution: comparison of two ways of evaluation—a case study from Europe

**DOI:** 10.1038/s41598-024-56403-9

**Published:** 2024-03-13

**Authors:** Klaudia Borowiak, Anna Budka, Marta Lisiak-Zielińska, Kinga Robaszkiewicz, Arlinda Cakaj, Tropikë Agaj

**Affiliations:** 1 Department of Ecology and Environmental Protection, Faculty of Environmental and Mechanical Engineering, Poznań University of Life Sciences, Wojska Polskiego 28, 60-637 Poznań, Poland; 2 Department of Construction and Geoengineering, Faculty of Environmental and Mechanical Engineering, Poznań University of Life Sciences, Wojska Polskiego 28, 60-637 Poznań, Poland

**Keywords:** Urban ecology, Sustainability

## Abstract

Visual pollution by outdoor advertisements (OAs) of the urban landscape in the old town of Gniezno city (Poland) was the main aim of the presented work. For this purpose, the part of Warszawska Street located near the old market was selected. The detailed objective of the work was to evaluate the effect of OA on the building perception and compare two different methods to check the possibility of their interchangeably potential. The city audit and public opinion about selected buildings were performed. The photographs with and without OAs were also analyzed. The statistical analysis was performed to find the significant differences between these two methods and define differences between groups of respondents. In general, the results revealed the strong effect of the building quality (condition and appearance of the building) on the final score of the building perception. This was valid for both evaluation methods. The significant differences between building perception with and without OAs and groups of respondents were found only for the building with good facade quality. While, in the case of low-quality buildings the perception of the urban landscape was low regardless of the presence of OAs. Moreover, we can primarily conclude that city audit and survey of public opinion can be used interchangeably.

## Introduction

In relation to social, environmental and cultural issues, the landscape has become one of the main topics for territorial sustainable development^1^. To create a suitable urban landscape, one of the factors that must be paid attention to is visual comfort, which includes the quality, color and brightness of the environment^2^. Landscapes are spatial–temporal systems which are hierarchically related to biotic, abiotic and anthropogenic elements. For landscape assessment, new methods have brought a definition that environment and landscape can be defined as an integrated conception^3^, but unfortunately, they are experiencing quality degradation which is already on the rise and the continued growth, especially in urban areas.

Advertising in the form of a poster appeared in Poland a little later than in Europe—in the nineteenth century. It began to be treated more and more professionally, but its real development took place in the interwar period, when a number of advertising agencies and specialized companies implementing posters in urban spaces were established. From this period has come a great many interesting advertisements, both in terms of content, but also in form. After World War II, neon signs and murals filling entire walls of buildings as advertisements were gaining popularity. Advertising took the form of social advertising carrying an important message to the public. Since 1989, advertising in Poland has been in line with trends in global market—creating positive images of the product and presenting an offer of a modern lifestyle. In the early 1990s, advertising in the media and public spaces was accepted by the public without serious objections. Today, however, our view of it is quite different^4^. The main problem in many Polish cities is both the number of advertising objects, but also their form (including wide variety of size, style and color as well as lack of coherence in the composition). This creates a sense of chaos and lack of spatial order. This problem is particularly relevant for areas located in the historical zones, where outdoor advertisements should be located thoughtfully^5^.

Over the years, outdoor advertising has progressed significantly, from paper signs to illuminating screens and interactive LED billboards. Advertisers use as much as possible of objective elements (facade, wall, urban furniture, permeability, closeness, skyline, readability, mixed and variety of applications, integration) and subjective elements (security, memories, sensory richness, sense of belonging and spatial identity, social solidarity) to rich the highest number of consumers^6^. An oversupply of OA may have the negative impact on public life by influencing cultural consumption patterns^7^, by the impact on mental health^8^, the landscape visual quality^9,10^, reduction of property values, affecting site identity, encourage consumption of unnecessarily, damage public places^6,11^, distraction of driver especially on major highways^12–16^, impaction on planning strategies^17^ and loss of cultural identity^7^. Moreover, lots of city governments do not find it easy to regulate OAs because they don't have any systematic methods to determine the amount of its presence as well as to measure the intensity of its impact on visual quality^18^.

Recently, visual pollution, including OAs, has become increasingly important^19^. However, in comparison to air or water pollution, for which there is a long legacy of investigation in the physical, biological and natural sciences, visual pollution mostly requires a legal (law), cultural, and physical study mostly related to urban areas^19–21^. Mainly in the mid-twentieth century, in terms of evolution, the term ‘visual pollution’ was recognized and researched^21^. Visual pollution is a muddled impact of messiness, disorganization and conquering different items and designs in the landscape like lighting features, street furniture^22,23^, media devices and different outdoor advertisements (posts, billboards, works and signs in additions to flags, etc.)^24^. These factors are generally noted in urban conditions and a certain environment as a result of the lack of control, wrong or lack of decisions, applications incompatible with performance, lack of legal requirements, and a lack of culture and education^19^. Each one of those components which a community finds unwanted, unpleasant, intrusive, and upsetting comes under visual pollution. Researchers engaged in assessments of the visual impact of natural landscapes have used the term visual pollution objects (VPO) to define the physical components that have the capacity may impede the view of valuable natural complexes or may contribute to the reduction of visual importance and to reduce the visual quality of the landscape^25^

For landscape physiognomy, visual pollution in the form of advertising is a threat^26^, because the increases of outdoor advertisement without aesthetic consideration can be considered as a form of spatial chaos, which means a phenomenon of destruction of the visual landscape^27^. The visual pollution induced by out-of-home advertising has been related to the encroachment of private command over open public spaces, impacting the city life flow^28,29^. Moreover, Chmielewski^26^ for their study purposes, define visual pollution as the closure of the human field of vision by the features of OAs, which is strong enough to disrupt the perceived character of a landscape. And this definition refers to the influence of OAs in the field of view of the observer in terms of occlusion and distortion of the landscape visual^30^.

The number of studies on assessing the visual attractiveness of landscapes is very large, and this is related to the great variety of used methods, assumed criteria or scales. The main aims and objectives of these methods are also very different. Most of the methods used both in Poland and in the world have focused on natural landscapes with elements of cultural landscapes. There are noticeably fewer methods dedicated to cultural landscapes, including urban areas or visual pollution objects^31^. Many of these methods are expert methods that do not take into account public participation. According to Gruehn and Roth^32^ local communities, very often perceive and evaluate the landscape differently than experts, hence there is a need for methods that take into account public perception. City audit is defined as a modular procedure for collecting and processing information on the social, economic and urban diversification of the city^33^. According to Czornik^34^, it is a modern attribute that gives the possibility of the spatial development of the city. The main goals of the city audit are determining the degree of compliance of the condition of the analyzed object with the established criteria, recognizing limitations and indicating directions of changes. This allows the focus the activity of city authorities on achieving social, spatial and economic attractiveness of the managed area, and contributing added value to the city.

Regarding visual pollution, the city audit is a procedure that permits the recognition and identification of zones with existing urban problems, checking the way of using the space in the zones and the prediction of their degradation in the future. In other words, the city audit is an assessment of the harmony of the parameters of an object with the audit standard. The novelty of present study was related to the comparison and selection of reliable city landscape quality method with the stress to OAs influence. We have compared the methods based on the expert knowledge (city audit) and general public opinion (survey), and as a result the conclusion of usefulness of city audit method instead of wide range public questionnaire was analyzed. Hence, the aim of the present study was to evaluate the potential visual pollution of the urban landscape and the historical streets in the old town of Gniezno City, Poland. The detailed objectives were as follows: to compare the evaluation of street visual pollution by city audit method and perception by public opinion (i); to evaluate the effect of OAs on street landscape perception (ii) and to indicate the possibility to use only city audit instead of time-consuming public opinion tests (iii).

## Results

### City audit and degradation map

**Figure 1:**
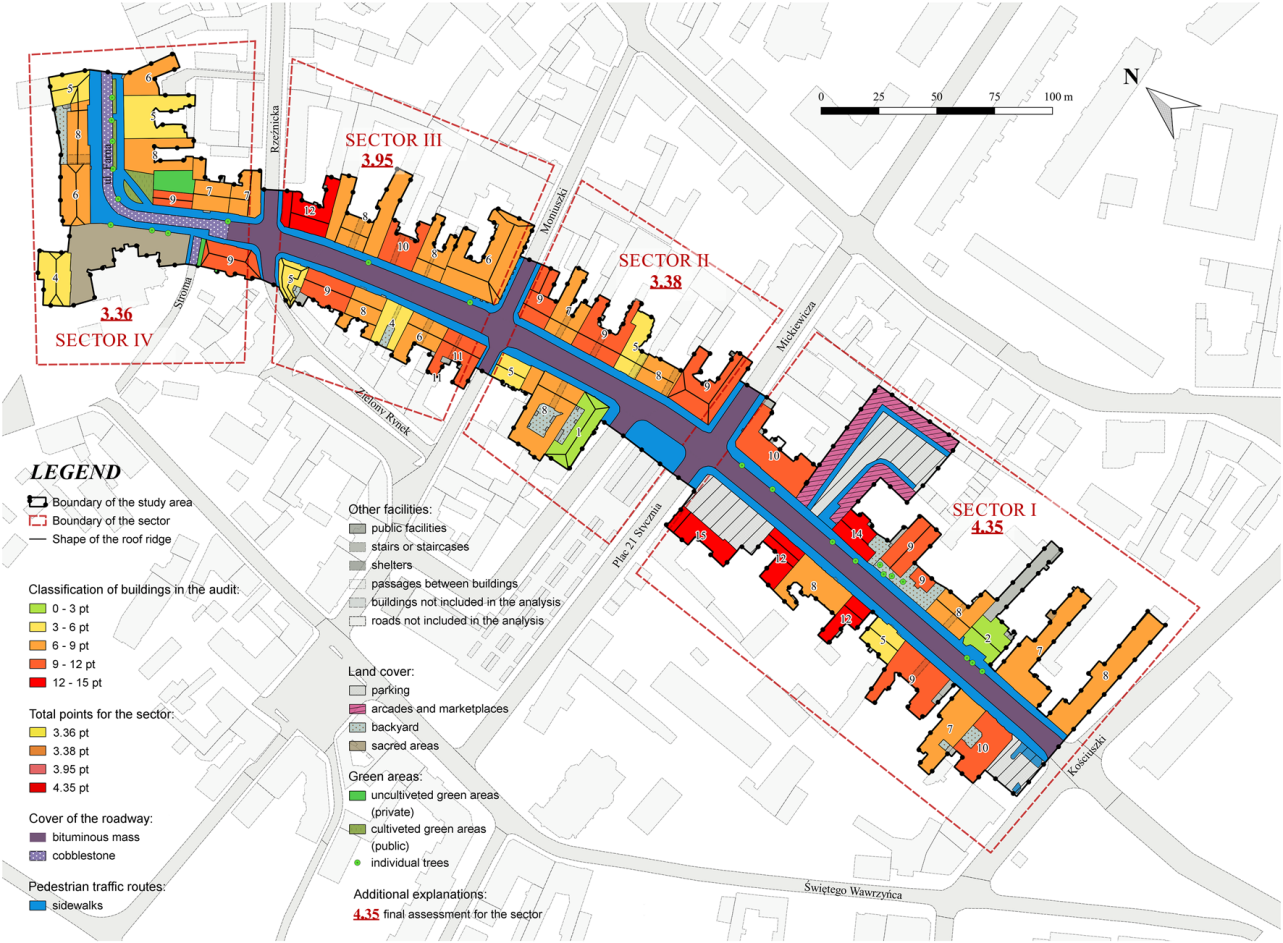
Degradation map of the individual buildings of Warszawska street (source: own study based on data from National Geodetic and Cartographic Resource).

The detailed analysis of individual building evaluation revealed the highest number of degradation points in the first category (the building condition of the facade, renovation of space and roads and pavements) was given to the parking lot at Plac 21 Stycznia Street No. 2. The space within the buildings at Warszawska Street No. 27, 29, 30A as 19 and 4, which received 7 points, is also characterized by high degradation. Buildings with a score of 6 or 5 look slightly better, as in case of Warszawska Street No. 1, 2, 3, 11, 16, 21, 26, 26A, 40, 34 and buildings at Farna Street No. 1, 2 and Kościuszki Street No. 54. The other spaces received from 0 to 4 points in total, so the degree of degradation is not extremely high. The scale of changes introduced by the advertisements themselves is the largest for the building located at Warszawska Street No. 16, which is as much as 9 points. Buildings on the street Warszawska Street No. 4, 6, 9, 13, 14, 15, 32, 34, 37, 38 received 5 or 6 points on the scale of transformation with advertisements. The remaining buildings have a low point rank, and the incidence of advertisements is moderately high there (Fig. 1).

The subject assessment of the degree of degradation of the street by outdoor advertisements was performed on individual objects of the urban tissue, such as buildings. Based on the assessment made for individual facades and surroundings, the results were classified into four specially designated sectors. The division into sectors was made in accordance with the emerging cross-streets and the character of the buildings.

**Figure 2:**
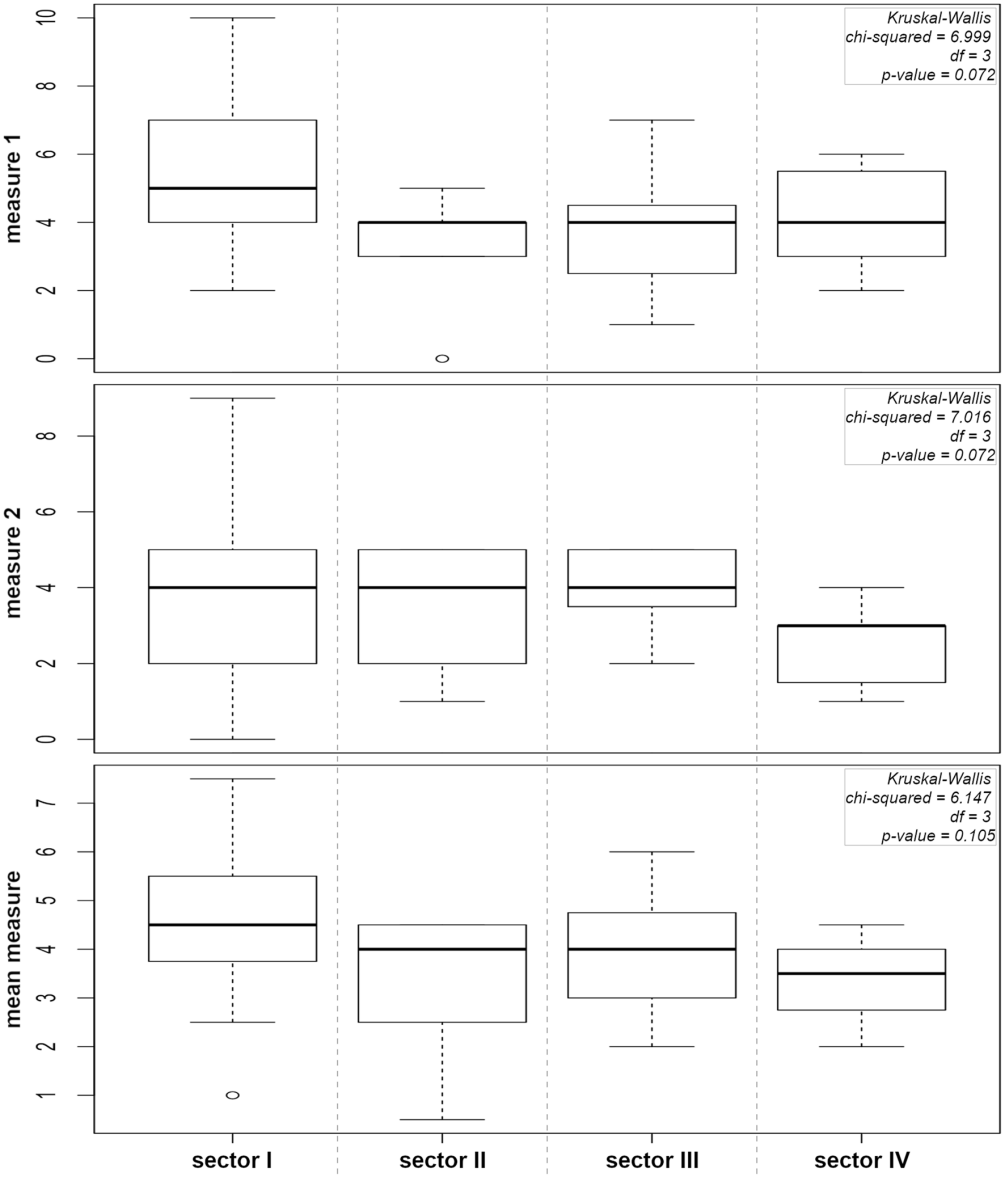
Average value of urban landscape degradation (measure 1, measure 2, mean measure) in certain sectors of Warszawska street. Chi-squared values and p-value are included for each measure.

The Kruskal–Wallis test for city audit in the sectors revealed the lack of significant differences between sectors for both measures. However, the detailed analysis showed some tendencies. The first sector was found with the highest values of both measures as well as the mean measure and with the highest range of observed values. In the case of the second sector measure 1 revealed the lowest range of values and the lowest mean value of all sectors, which means that this sector has the best quality of the building facade. The level of measure 2 in this case was at a similar position in sector 1, with a similar range of values. Sector 3 revealed a relatively small range of values for both measures. Sector 4 was found with the lowest measure 2 value and the small range of values. This sector revealed the lowest measure 2, which means that the negative effect of OAs was the lowest. The confirmation of this observation was noted in mean measure, where the highest value was recorded for sector 1 and the lowest for sector 4 (Fig. 2).

**Figure 3:**
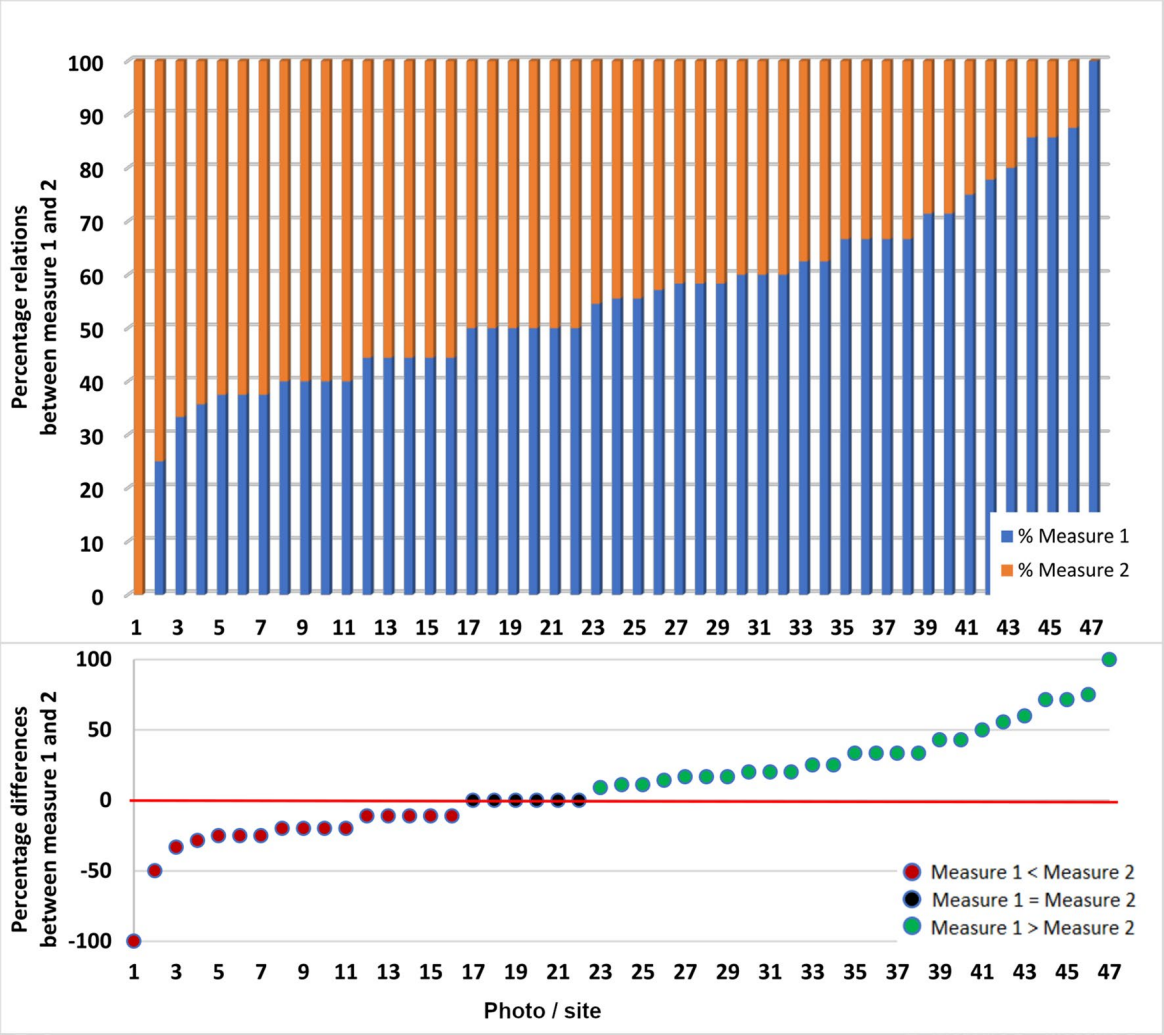
The ascending order of the difference between particular measures 1 and 2.

The additional analysis was performed to determine the effect of certain measures on the particular building measure. Based on the results of differences between measures 1 and 2 we can conclude that building facade quality had a stronger effect on the final building evaluation (Fig. 3).

### Questionnaire results

The analysis of individual respondent reliability was performed using the ICC2 (2.1) coefficient in the random ANOVA model for dependent groups. This model was found to be highly statistically significant F = 237.6 (p < 0.000001). Moreover, for a single respondent, the value of the ICC coefficient (2,1) = 0.481 with an appropriate 95% confidence interval (0.286; 0.793). This means that each of respondents evaluated each photograph of buildings with the highest reliability and repeatability of the tendency within an individual evaluation.

The second ICC coefficient (2, 259) = 0.996 with a confidence interval (0.990; 0.999) proves the high consistency of the results obtained from 259 respondents. The high value of this coefficient proves that the judges' ratings were consistent for the individual photos. This may reflect a large variance between subjects (photos) and a small variance between judges' ratings.

The analysis of responses for photographs with and without OAs revealed the variability of significance levels between analyzed social groups. Generally, there is no difference between evaluation by women and men. However, we noticed that the first three photographs' assessments with and without changes varied between these two groups. Moreover, the analysis of significant differences between photographs with and without OAs evaluated by the women group separately indicated the significant difference only for the first two photographs. Similar tendency was noticed for the men group, the two youngest age groups, the group with a high school education and all places of residence. The last two photograph evaluations in terms of the effect of OAs did not vary significantly in almost every social group (Table 1).

**Table 1:** The Kruskal–Wallis test within the social groups indicated significant differences between building with and without OAs (*significant difference at the level ≤ 0.01, *ns* not significant).

Parameter	All photos	A–A′	B–B′	C–C′	D–D′
Gender					
All genders gropus	ns	*	*	*	ns
Women	*	*	*	ns	ns
Men	*	*	*	ns	ns
Age					
All age groups	ns	*	*	*	ns
< 25	*	*	*	ns	ns
25–45	*	*	*	ns	ns
45–65	ns	ns	ns	ns	ns
> 65	ns	ns	ns	ns	ns
Profession					
All professional groups	ns	*	*	*	ns
Vocational education	ns	ns	ns	ns	ns
Secondary education	*	*	*	ns	ns
High education	*	*	*	*	ns
Residence					
All residence groups	ns	*	*	*	ns
Gniezno city	*	*	*	ns	ns
Gniezno county	*	*	*	ns	ns
Outside from Gniezno county	*	*	*	ns	ns

**Figure 4:**
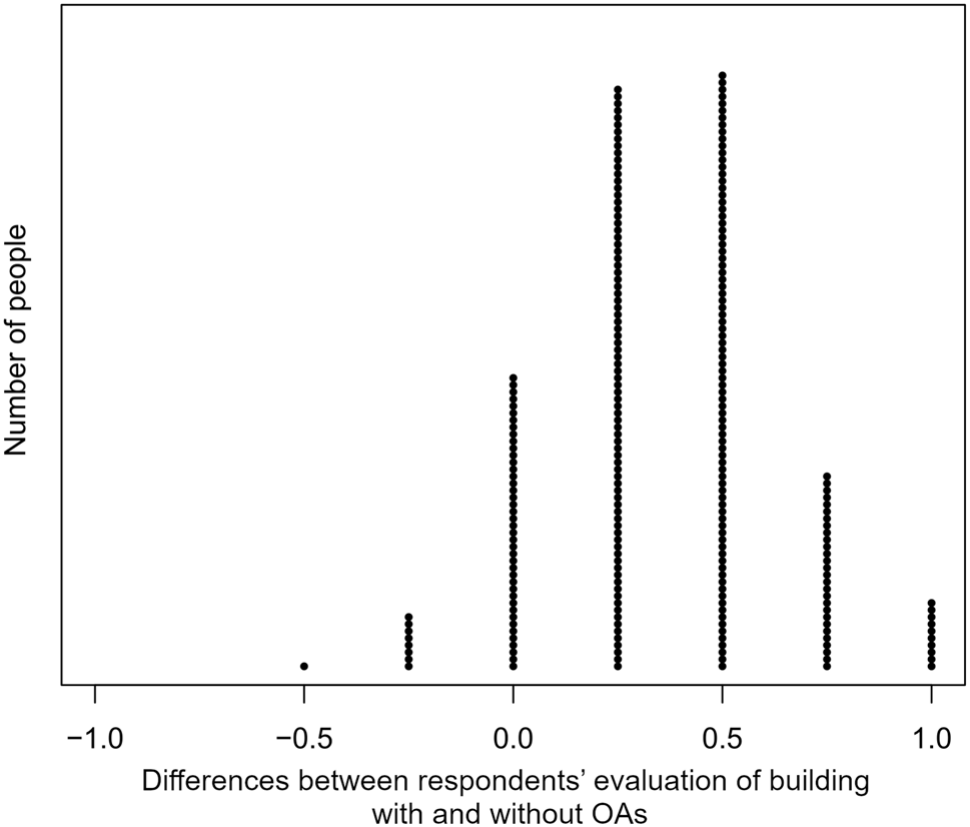
Differences between photographs respondents’ evaluation of building with and without outdoor advertisements (one point means one person and values above zero indicated a higher rating for buildings after the removal of OAs).

Generally, we can find the following tendency for all respondents’ evaluation—higher scores of photographs without OAs. Moreover, the average point difference between photographs respondents’ evaluation of building with and without OAs mostly varied between 0.25 and 0.5 (Fig. 4).

**Figure 5:**
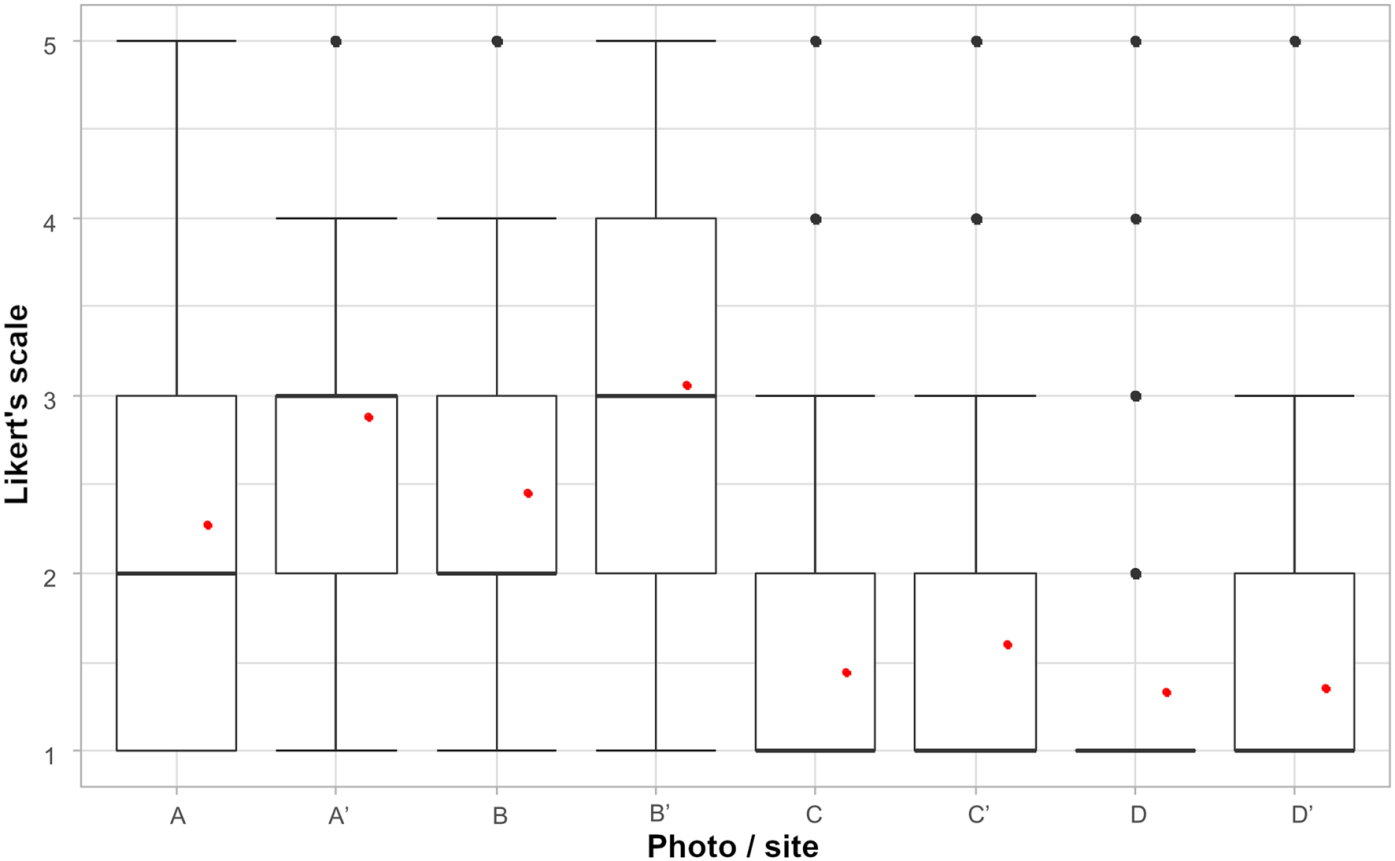
Mean value of rating by public opinion photographs with and without outdoor advertisement (plus minus standard deviation, black dots—outliers, bold black line—median, red dots—mean).

The detailed analyses of selected photographs with and without graphical modifications confirmed the increase in public acceptance of the building quality. Photograph A and B were found with the relevant increase in the level of public acceptance for the building and OA quality. Photograph D was found to have a very low level of values of public ratings, while after graphical modification and OAs removal an increase in values of public acceptance was observed. While in the case of photograph C, the removal of OAs from the building did not affect public opinion (Fig. 5).

### Relations between public perception and audit method

Comparison between city audit results and ratings of respondents was based on the six photographs without graphical modification. The mean values of measures 1 and 2 and the averages were analyzed in relation to the mean ratings of respondents. The mean values of certain measures were ranged between 2–5 and 2–9 points, respectively measure 1 and 2. While the mean value of respondents’ ratings ranged between 1.333 and 3.096 (Table 2). For these 6 photographs the correlation coefficients between mean values rating and measure 1, 2 and mean value were − 0.612, − 0.351 and − 0.495, respectively. It is crucial to remember that the attractiveness scale is the opposite in these two methods of attractiveness evaluation, which explains the negative correlations.

**Table 2:** Measure 1, 2 and mean measure values and the mean value of respondents’ rating.

Photo	Measure 1	Measure 2	Mean measure	Mean value of respondents’ rating
A	2	4	3	2.434
B	4	4	4	2.635
C	5	9	7	1.452
D	4	6	5	1.333
E	4	2	3	1.703
F	3	5	4	3.096

**Figure 6:**
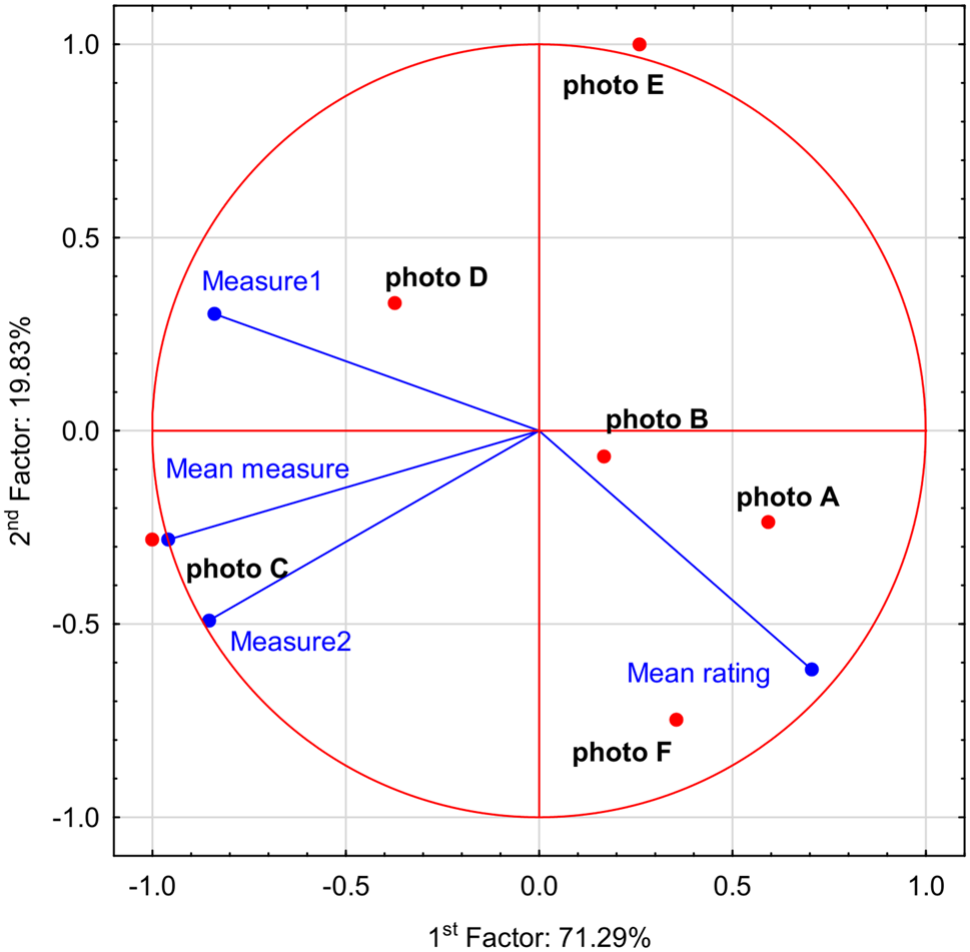
Principal component analysis of measure 1 and 2, mean measure and mean ranting of public perception.

Principal component analysis may be able to explain over 90% of the total variability of analyzed data. Audit measures 1 and 2 revealed a positive correlation between both of them, as well as to mean value of the measure. A negative relation was noted between the mean value of respondents’ rating and all measures. Moreover, the strongest negative correlation was observed for measure 1 and the mean value of respondents’ ratings. The highest mean rating of public opinion was recorded for photographs A i F, with the simultaneous lowest measures values. These results indicated the lowest negative effect of OAs and building quality. The opposite relation was recorded for photographs C and D (Fig. 6).

## Discussion

Improving city perception is one of the most important aims of urban managers. It is one of the key factors affecting the well-being and quality of life of residents, but also potential tourists^35^. Gniezno is a city with a strong historical value, including buildings, especially in the city center. Therefore, it is essential that the perception of the city will not be distorted by visual pollution, which can lead to negative consequences, such as loss of identity^19^.

The city audit of Warszawska Street revealed that the most important in the general perception of the building is the quality of the facade and area in the front of the building. Building facades are a fundamental element of the urban landscape, which is affected by many factors, such as color, shape, texture, and material^6^. The visual qualities offered along the street are also very important for pedestrians—the number of doors, windows, columns, storefronts, display details, signage, and decorations are significant for their perception^36^. In addition, their perception also depends on external factors—weather and lighting conditions^6^. According to Yilmaz and Sagsöz^19^, all these elements, combined with a monotonous and dense structure with a disproportionate number of floors, themselves create visual pollution, which was also confirmed by our research. Moreover, the negative perception is further intensified by other elements, including signage on buildings or large billboards^19^.

To receive the precise relation between OAs and human city landscape perception, as well as to building quality, the questionnaire with photographs of selected buildings was conducted. Moreover, the photographs modifications were also performed, and OAs were removed from selected photographs. Hence, the comparison of results with and without OAs was possible. The statistical analysis of the results revealed a significant difference with and without OAs with a positive perception of the latter one, which is consistent with previous studies. According to a study conducted by Wakil et al.^25^, outdoor advertisements and signage, as well as dilapidated buildings, are considered the biggest contributors to visual pollution. Similar results were obtained by Nami et al.^37^, as the majority of respondents indicated that visual pollution affects the beauty, attractiveness, vitality and vibrancy of urban spaces. However, it is worth noting that in our study, most of the OAs were located near historic townhouses, which may have increased negative public attitudes. As Boştină-Bratu et al.^38^ research showed, the public's evaluation of OAs depends on a number of factors, including the type of OAs, location, colors, or even the message of the OAs. For example, billboards used to cover a construction or renovation site in the city are much more acceptable compared to OAs placed on or near churches, as well as in the old market and the city center^38,39^. Some differences in opinion were also found between the respondents, such as women and men, as well as different age groups. Similar results were observed in a study of the color content and cumulative area of outdoor advertising billboards on the visual quality of urban streets. Adam et al.,^40^ observed gender differences only for selected images in Kuala Lumpur (Malaysia)—men showed greater acceptance of OAs. In addition, the responses of people over 26 years old differed from younger respondents, suggesting that older adults were more sensitive than younger adults. Also, Nawrocki^39^ showed that older people in Rybnik (Poland) have lower acceptability of outdoor advertising. In addition, a survey of residents in Rybnik on perceptions of advertising showed that those with a higher education felt that outdoor advertising did not contribute to the city's beautification and was less likely to approve of it^39^. However, we have also noticed that the poor quality facade of the building significantly influenced the building perception, and the location of OA did not influence the perception of these buildings. The obtained results are indirectly supported by the research of Nami et al.^37^, as this study showed that for respondents in public spaces, restoration and beautification of historical buildings and monuments and proper shaping of space elements, are more important than OAs.

Most urban landscape assessments are based on a single level of analysis using a single methodology. To assess the quality of an urban landscape, a selection of methodologies has to be combined in order to generate a reasonable result^41^. Our investigations proved that the city audit is comparable to human perception as well as the quality of the building is crucial to the overall evaluation of the building's appearance and general city landscape. Moreover, we can also conclude that sophisticated OA might not visually pollute the urban landscape.

Although the research conducted has provided new results some potential limitations should be noted. The limitation of the current research was related to the number of photographs presented in the questionnaire. The number of photographs also limited the possible number of combinations of building type and condition and type of OAs. However, in our research due to simplify and shorten the survey, the number of photographs has to be reduced to achieve a representative number of questionnaires. Another limitation of our research is our case study focused on certain type of buildings (historical). Hence, the results of similar investigations for modern buildings might be different. The future research should include different types of buildings characteristic of certain architectonical style.

## Conclusions

The visual pollution of the historical center of Gniezno city has been here found due to the poor quality of the building and additional outdoor advertisement locations.

The most influencing factor on building perception was the facade and surroundings of the building.

The evaluation of the building without the outdoor advertisements was higher in comparison to those with outdoor advertisements. However, in the case of buildings with poor quality facades or low architectural standards the outdoor advertisement was not significant in the total building perception.

The city audit method was quite similar to the public opinion received during the questionnaire; hence we can assume that these two methods might be used interchangeably.

## Materials and methods

### Study area

Gniezno is a city located in the in west-central Poland, in the eastern part of Wielkopolskie Region. The city covers an area of 41 km^2^ and is inhabited by almost 65,000 people^42^. The spatial character of the city has been formed by several factors, but the most important ones are the Gniezno cathedral (the most important component and spatial dominant), the historic midtown with its compositional (view) axes and railway lines.

**Figure 7:**
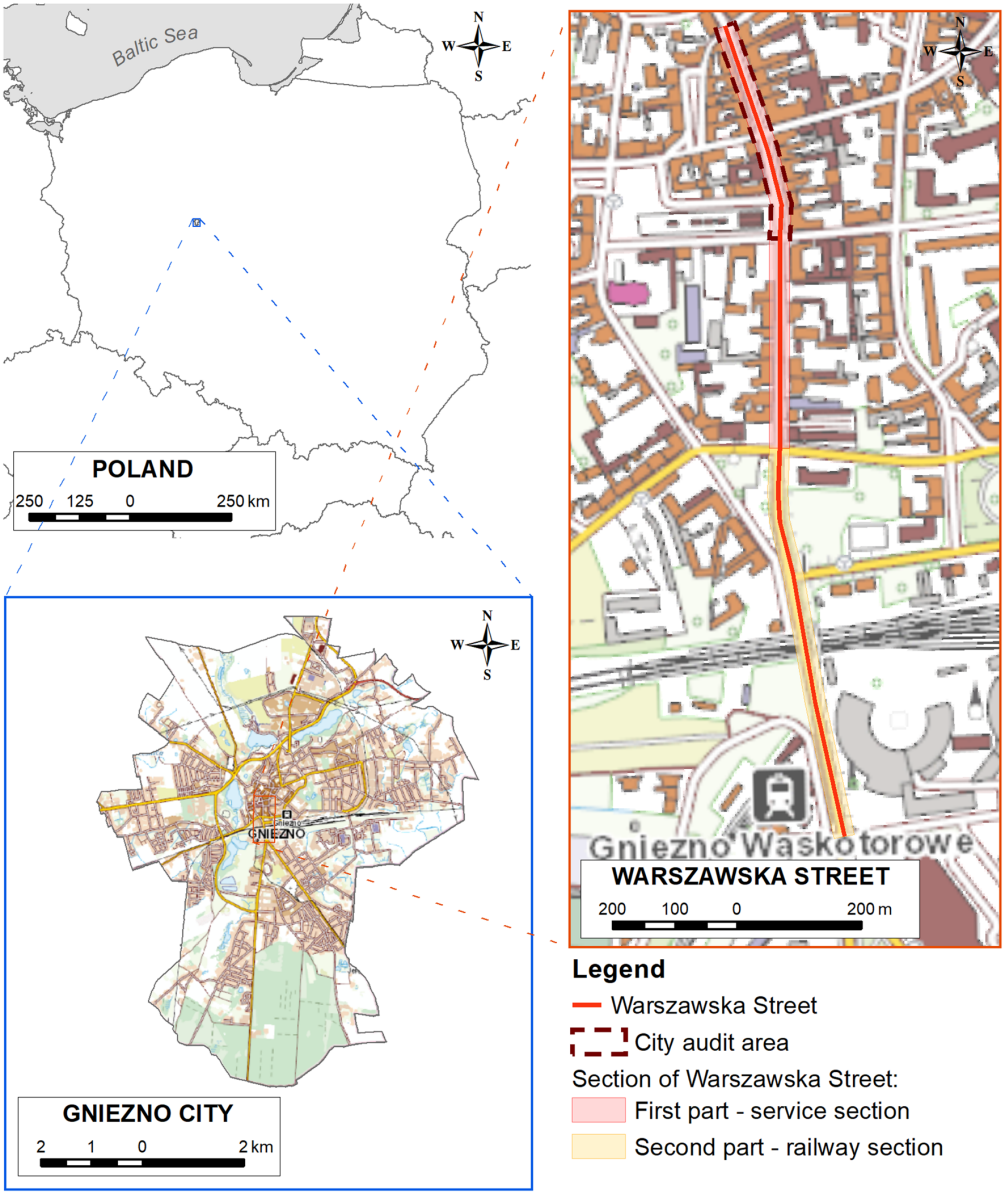
Location of Gniezno town and Warszawska Street (source: own study based on data from National Geodetic and Cartographic Resource).

**Figure 8:**
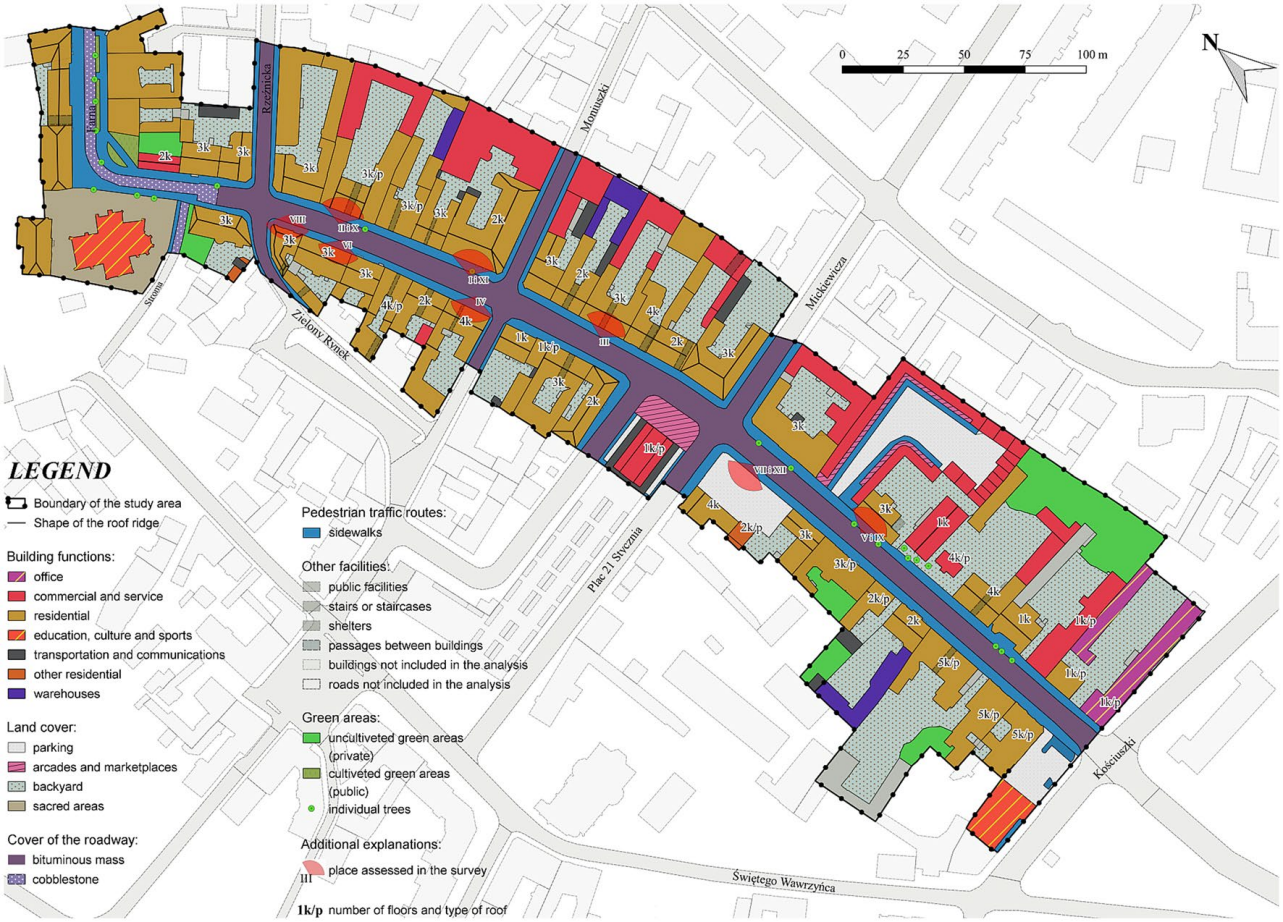
Characteristics and division of Warszawska Street in Gniezno (source: own study based on data from National Geodetic and Cartographic Resource).

Warszawska Street, one of the oldest streets in Gniezno, was selected as the research area. The street can be divided into two separate sections with different morphologies. The first of them measures 490 m with an average width of approx. 8.3 m. It is mainly of a service character, because commercial and service premises are located in most of the buildings on the ground floor. Flats are located on the other floors of the buildings. Most of the buildings are historic tenement houses owned by private people. This part is under the protection of the Conservator of Monuments. The second part of Warszawska Street is a section of a different character. The length of this section is 430 m, and the average width of the road is approx. 8.7 m. The dominant buildings in this section are non-functioning railway transport facilities, a park and an education building (Fig. 7). Only part of this section of Warszawska Street is protected by the conservator. Warszawska Street is located in an area where there is a significant deficit of housing resources and a low standard of living. Small commercial activities are mainly carried out there (Fig. 8).

### Methodology

**Figure 9:**
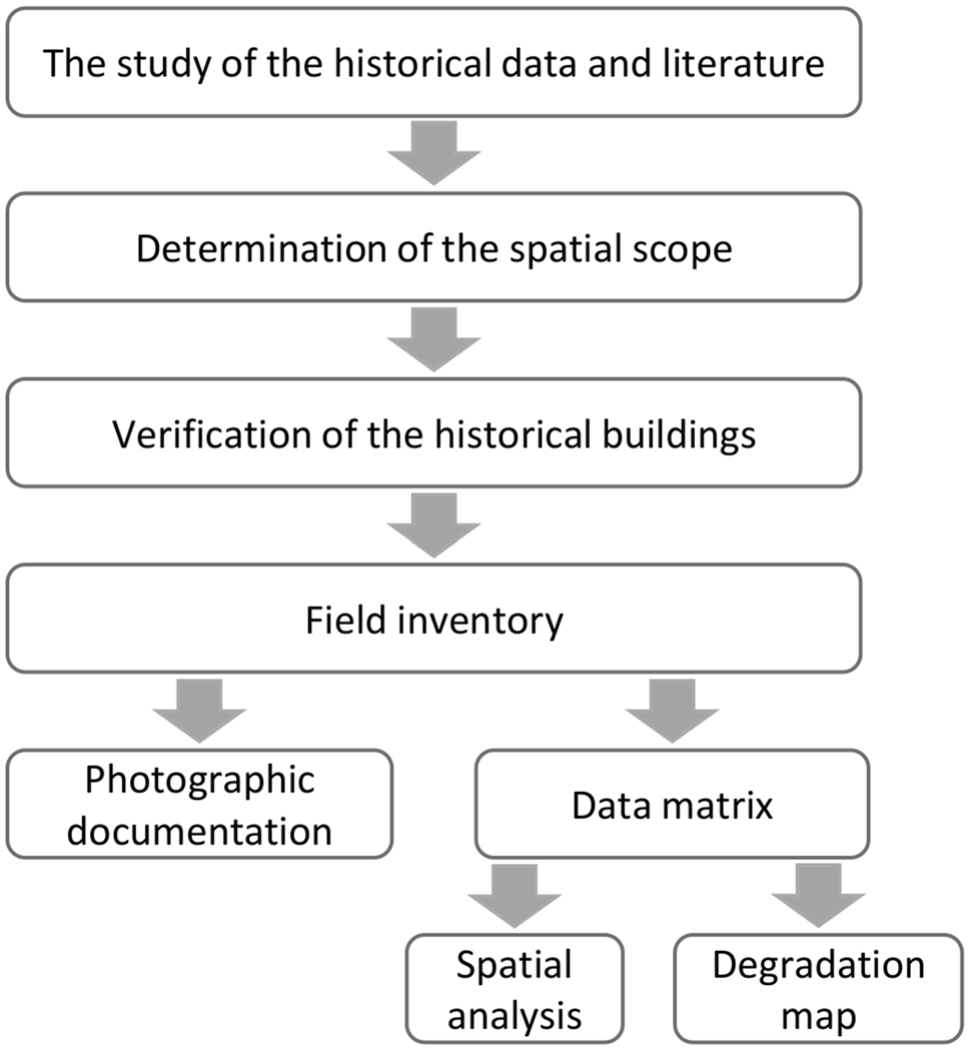
Methodological scheme in the city audit procedure.

The city audit is an assessment of the perception of the quality of urban space with the outdoor advertisements, which disrupts the functions of places. The methodology of city audit consisted of several stages that were dependent on each other (Fig. 9).

The study of the historical data and literature was made for collecting information about the history of Warszawska Street in Gniezno and about social, economic and spatial functions of the street in the past and at present. The strategic and planning documents for the city of Gniezno, as well as the historical documents from the end of the nineteenth century, were analyzed.

The spatial scope was distinguished based on historical data and literature. According to the methodology proposed by^43^, the audit area should be characterized by morphological, social and functional homogeneity. Therefore, the section of Warszawska Street from Mickiewicza Street to Gnieźnieńskich Olimpijczyków Round was excluded from the study, which differs in functional and social terms from the first section and is not morphologically related to the rest of the study area.

It was found that the majority of buildings in the studied area are under the protection of the conservator. During the field inventory each building was analyzed, and a series of photos were taken. The focus was on outdoor advertisements and therefore on various visual objects. The field inventory also included the location of the other elements of the urban infrastructure on Warszawska Street, such as: type of green areas, pedestrian roads (sidewalks), architectural elements and roadways.

The data matrix was based on the previous stages and is a special form which includes the assessed features and the assessed spatial units—buildings along Warszawska Street. The scale of the assigned points was established, ranging from 0 to 4:

- 0—no problem (elements degrading the analyzed space) or its importance is insignificant in the scale of the urban unit,
- 1—insignificant occurrence of the problem (the problem concerns less than 10% of the analyzed street),
- 2—occurrence of the problem (the problem concerns 10–40% of the analyzed street),
- 3—increasing occurrence of the problem (the problem concerns 40–80% of the analyzed street),
- 4—particular increase of occurrence of the problem (the problem concerns above 80% of the analyzed street).

The general research activities in the audit procedure covered the social and spatial systems, where each of which is independent and has its own unique internal connections. The scale of the degradation of space by outdoor advertisements was assessed in several categories. The categories were divided into two measures. Measure 1 is related to the quality of the building and surroundings, while measure 2 is describing the effect of outdoor advertisement on the building.

Measure 1 components:

- A—quality and condition of the building facades,
- B—general quality and condition of the space around the building (including the type of green areas, architectural elements and additional elements),
- C—quality and standard of roads and pavements,

Measure 2 components:

- D—the need to modify the commercial and service information in the form of changing the visualization:
  - D1—the presence of a sign that reduces the proper visual perception of the facade and place,
  - D2—the presence of an advertising board that reduces the proper visual perception of the facade and place,
  - D3—the presence of an advertising object that reduces the proper visual perception of the facade and place.

The result of the city audit is the level of the disturbance of the harmony of space determined based on outdoor advertisements placed in a given place on the building's facade along Warszawska Street. A synthetic map of urban landscape degradation is a graphic form of city audit interpretation^43^.

Additionally, a survey was carried out in order to find out about the local opinion on the perception of the space along Warszawska Street. The survey consisted of 12 photos of the building facade. The photos were taken from 8 places on the section of Warszawska Street, showing various types of facade conditions as well as the location and character of outdoor advertisements. All selected buildings had a similar architectural style. Moreover, photos of 4 buildings were significantly modified (Supplementary Table S1). They were presented twice: in the current state—with outdoor advertisements, and after graphical modification—with complete removal of outdoor advertisements. Original and modified photos did not follow each other in the online survey, and after evaluating one of the photos, it was also impossible to return to the previous photo. Each photo was rated by the respondents on a Likert’s scale from 1 to 5, where 1 point meant that the respondents perceived a given landscape as not very attractive and 5 points meant that the place was perceived as very attractive. Moreover, to evaluate the effect of certain groups of respondents’ data such as age, gender, education level and place of residents were also asked in the questionnaire. The questionnaire had a form of fully anonymous and has been provided within direct personal distribution—paper form (12%) and online questionnaire (88%). The results of 259 responses have been achieved.

### Statistical analysis

The results of the city audit were analyzed with the Kruskal–Wallis test, as well as visual data presentation with the indication of average, medians and outliers was presented.

The analysis of questionnaire results was performed with the aid of several statistical methods. For the analysis of the reliability and compatibility of the respondent's answers the two tests were performed. Due to the nature of the research (each respondent assessed all the photos), the second model of the intra-class correlation was used. To check the reliability of each respondent the ICC2 (2.1) coefficient was determined in the random ANOVA model for dependent groups. To check the consistency and compatibility of all groups of respondents, the intraclass correlation coefficient (ICC) was used for k = 259 people, measuring the strength of the ICC judge's reliability (2.1) and the degree to which the observers' assessments were consistent with ICC (2.259).

To investigate the tendency of the response of the effect of OA the distribution of mean differences between with and without photographs was conducted.

For the comparison of the answers related to photographs with and without OAs of certain groups the Kruskal–Wallis test was performed. Then the visual data presentation with the indication of mean, medians, standard deviation and outliers were presented.

The comparison of city audit and perception of city landscape by public opinion was performed with the aid of Pearson linear correlation coefficient and principal component analysis.

### Supplementary Information

Supplementary Table 1.

## Data Availability

All data included in this study are available upon request by contact with the corresponding author.
