# Supplementary material for: Urban visual pollution: comparison of two ways of evaluation—a case study from Europe
Source: Sci Rep. 2024 Mar 13;14:6138. doi: 10.1038/s41598-024-56403-9 (PMC10937664; doi:10.1038/s41598-024-56403-9)
Supplement: Supplementary file 1 — Supplementary Table 1. [file 41598_2024_56403_MOESM1_ESM.pdf]

Supplementary Table S1. Photographs presented in the questionnaire with (first column) and without OA (second column)

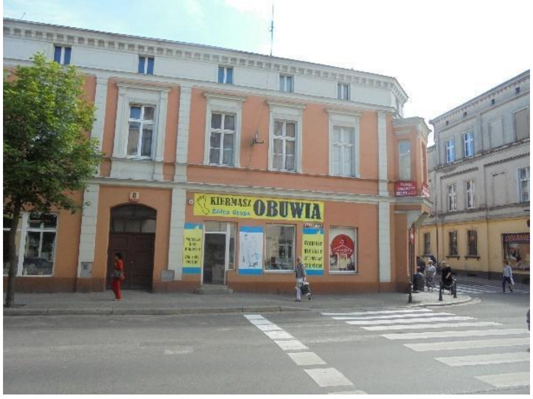

Photograph **A**

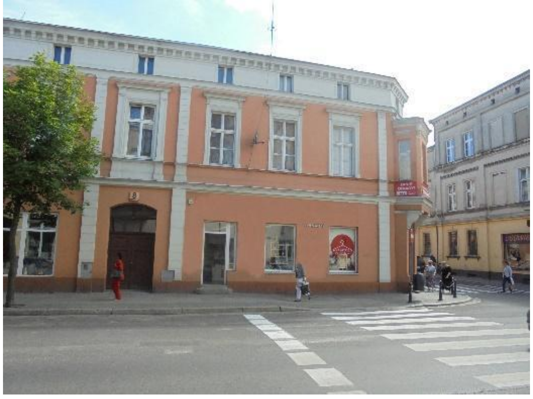

Photograph **A'**

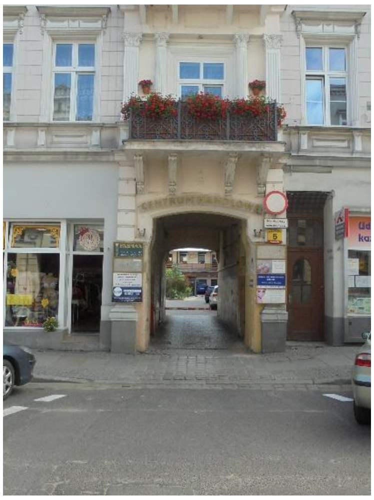

Photograph **B**

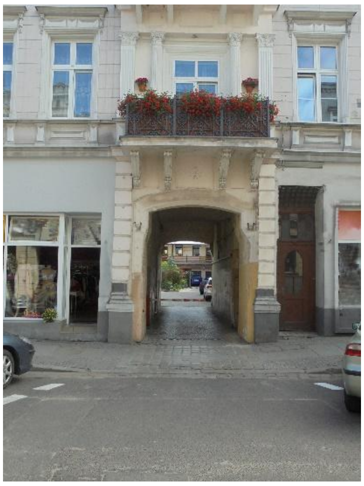

Photograph **B'**

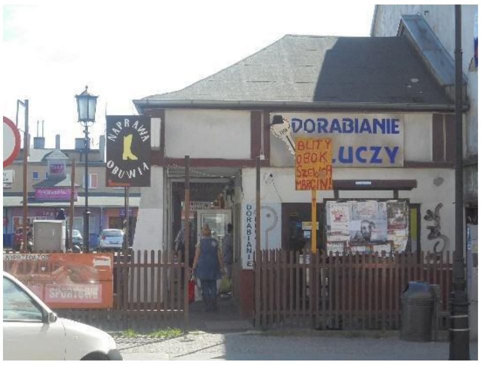

Photograph **C**

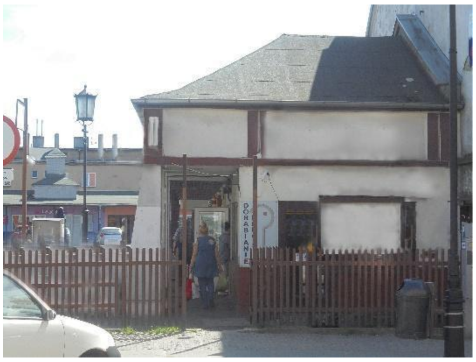

Photograph **C'**

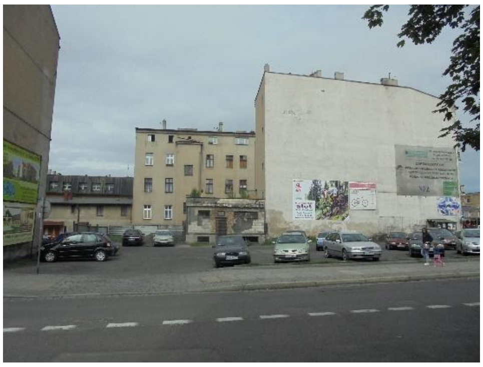

Photograph **D**

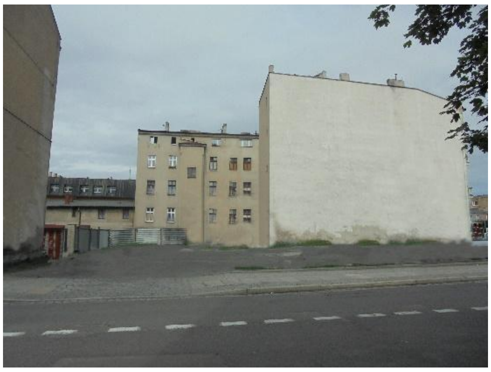

Photograph **D'**

Borowiak et al. (2024) Urban visual pollution: comparison of two ways of evaluation – a case study from Europe
